# Human Cortical Serotonin 2A Receptor Occupancy by Psilocybin Measured Using [^11^C]MDL 100,907 Dynamic PET and a Resting-State fMRI-Based Brain Parcellation

**DOI:** 10.3389/fnrgo.2021.784576

**Published:** 2022-01-20

**Authors:** Frederick S. Barrett, Yun Zhou, Theresa M. Carbonaro, Joshua M. Roberts, Gwenn S. Smith, Roland R. Griffiths, Dean F. Wong

**Affiliations:** ^1^Center for Psychedelic and Consciousness Research, Department of Psychiatry and Behavioral Sciences, Johns Hopkins University School of Medicine, Baltimore, MD, United States; ^2^Department of Neuroscience, Johns Hopkins University School of Medicine, Baltimore, MD, United States; ^3^United Imaging Intelligence, Shanghai, China; ^4^Division of Geriatric Psychiatry and Neuropsychiatry, Department of Psychiatry and Behavioral Sciences, Johns Hopkins University School of Medicine, Baltimore, MD, United States; ^5^Division of Nuclear Medicine and Molecular Imaging, Russell H. Morgan Department of Radiology and Radiological Sciences, Johns Hopkins University School of Medicine, Baltimore, MD, United States; ^6^Departments of Radiology, Psychiatry, Neurology, and Neuroscience, Mallinckrodt Institute of Radiology, Washington University in St Louis, St. Louis, MO, United States

**Keywords:** psychedelics, neuropsychopharmacology, psilocybin, PET, fMRI, resting state, receptor occupancy, MDL 100, 907

## Abstract

Psilocybin (a serotonin 2A, or 5-HT_2A_, receptor agonist) has shown preliminary efficacy as a treatment for mood and substance use disorders. The current report utilized positron emission tomography (PET) with the selective 5-HT_2A_ receptor inverse agonist radioligand [^11^C]MDL 100,907 (a.k.a. M100,907) and cortical regions of interest (ROIs) derived from resting-state functional connectivity-based brain parcellations in 4 healthy volunteers (2 females) to determine regional occupancy/target engagement of 5-HT_2A_ receptors after oral administration of a psychoactive dose of psilocybin (10 mg/70 kg). Average 5-HT_2A_ receptor occupancy across all ROIs was 39.5% (± 10.9% SD). Three of the ROIs with greatest occupancy (between 63.12 and 74.72% occupancy) were within the default mode network (subgenual anterior cingulate and bilateral angular gyri). However, marked individual variability in regional occupancy was observed across individuals. These data support further investigation of the relationship between individual differences in the acute and enduring effects of psilocybin and the degree of regional 5-HT_2A_ receptor occupancy.

## Introduction

Psilocybin is a psychedelic drug that has shown preliminary efficacy as a treatment for depression and anxiety (Carhart-Harris et al., 2016a, 2021; Griffiths et al., 2016; Ross et al., 2016; Davis et al., 2021) as well as substance use disorders (Johnson et al., 2014, 2017; Bogenschutz et al., 2015). However, there is a high degree of between-subject variability in the subjective effects occasioned by psilocybin and other psychedelic drugs. For instance, individuals vary in the degree to which they encounter peak or mystical experiences during the acute effects of psilocybin (Griffiths et al., 2011; Barrett et al., 2015; Barrett and Griffiths, 2017), and a modest percentage of individuals experience fear and anxiety during the acute effects of psilocybin (Griffiths et al., 2006, 2011; Studerus et al., 2011). Also, the degree of observed treatment response after psilocybin therapy (including one to three doses of psilocybin) can vary widely between patients with mood disorders (e.g., Carhart-Harris et al., 2016a,b).

A number of recent functional magnetic resonance imaging (fMRI) studies have shown that individual differences in acute subjective effects of psychedelics, such as visual imagery (Carhart-Harris et al., 2016b; Kaelen et al., 2016) and degree of peak experiences described as “ego-dissolution” (Carhart-Harris et al., 2014) tend to covary with individual differences in regional brain activity and connectivity. Preliminary evidence suggests that behavioral outcomes of psychedelic experience (Sampedro et al., 2017) and clinical outcomes from psychedelic therapy (Carhart-Harris et al., 2017; Roseman et al., 2017; Mertens et al., 2020) may also be associated with changes in regional brain activity and connectivity that persist after acute drug effects have subsided. While these studies are critical to understanding the underlying neurobiology of psychedelic experiences, they are limited in terms of identifying the mechanisms that determine individual differences in these experiences. For instance, although acute alteration of visual system activity may cause hallucinations (Kometer and Vollenweider, 2018), this activity does not explain why this might happen to a different degree in different individuals.

The primary molecular mechanism of action of psychedelics, such as psilocin (the active metabolite of psilocybin), is understood from preclinical literature to be 5-HT_2A_ receptor agonism (Halberstadt, 2015; Nichols, 2016). Human behavioral (Vollenweider et al., 1998; Carter et al., 2005, 2007; Quednow et al., 2012; Kraehenmann et al., 2017) and neuroimaging (Kometer et al., 2012, 2013; Preller et al., 2016, 2017; Barrett et al., 2017) studies using antagonist challenges with ketanserin (a 5-HT_2A/2C_ antagonist) support a necessary role of the 5-HT_2A_ (and potentially 5-HT_2C_) receptor in the acute subjective, cognitive, and neural effects of psilocybin and other such as LSD. Recent molecular imaging with positron emission tomography and the 5-HT_2A_ partial agonist ligand CIMBI-36 have demonstrated an association between plasma psilocin levels, neocortical 5-HT_2A_ occupancy, and subjective effects of psilocybin (Madsen et al., 2019). 5-HT_2A_ gene expression maps have been associated with psilocybin-induced changes in global connectivity of sensorimotor cortical regions (Preller et al., 2020), and degree of overall neocortical 5-HT_2A_ occupancy has been associated with duration of psychedelic effects as well as magnitude of mystical experience scores (Stenbæk et al., 2021). However, the regional distribution and degree of 5-HT_2A_ receptor occupancy by psychedelics has yet to be demonstrated in humans, with previous studies reporting whole-neocortex 5-HT_2A_ binding (Madsen et al., 2019; Stenbæk et al., 2021).

The current open-label pilot study used positron emission tomography (PET) with the radiotracer [^11^C]MDL 100,907 (a.k.a. M 100,907) to determine the regional occupancy of 5-HT_2A_ receptors in the human brain after oral administration of psilocybin (10 mg/70 kg). [^11^C]MDL 100,907 is an inverse agonist (Weiner et al., 2001; Aloyo et al., 2009) that binds to 5-HT_2A_ receptors in a wide range of cortical and subcortical regions (Lundkvist et al., 1996; Gründer et al., 1997; López-Giménez et al., 1998; Hall et al., 2000; Kakiuchi et al., 2000; Talvik-Lotfi et al., 2000; Wong et al., 2008; Zhou et al., 2010), exhibits favorable test-retest reliability of 7-11% difference between tests (Talbot et al., 2012), is not significantly affected by changes in endogenous serotonin (Talbot et al., 2012), and is highly selective for the 5-HT_2A_ receptor (Johnson et al., 1996), with greater affinity and specificity than 5-HT_2A_ receptor antagonist ligands (including the widely used ketanserin and altanserin) (López-Giménez et al., 1997, 1998).

This pilot study was conducted to test the hypothesis that [^11^C]MDL 100,907 binding to 5-HT_2A_ receptors will decrease after oral administration of psilocybin in a broad range of cortical regions, including default mode network, primary sensory, and attention regions that were shown to have altered activity and functional connectivity in fMRI studies of classic hallucinogens (Carhart-Harris et al., 2012, 2014, 2016b; Barrett et al., 2017; Preller et al., 2017, 2020; Sampedro et al., 2017; Müller et al., 2018; Schmidt et al., 2018; Mason et al., 2020). This binding decrease is interpreted as 5-HT_2A_ receptor occupancy by psilocin, the active metabolite of psilocybin. 5-HT_2A_ receptor occupancy was estimated in ROIs derived using a data-driven approach (Craddock et al., 2012) from resting-state functional connectivity data that were measured in each of the study volunteers before PET or drug administration procedures were performed. This approach generates a study-specific brain parcellation with ROIs that show functional homogeneity, and allows for interrogation of 5-HT_2A_ receptor occupancy in volunteer-specific ROIs that are relevant in light of the growing literature showing regional differences in brain activity and connectivity during the acute and also enduring/post-acute effects of psilocybin and other psychedelic drugs.

## Methods

### Participants

The current study enrolled six healthy individuals, each with at least 25 lifetime exposures to a classic (5-HT_2A_ agonist) psychedelic drug. Two individuals (one female, one male) dropped out before any PET or drug administration procedures. The remaining four volunteers (2M/2F; mean age = 28 [27-30], all Caucasian) completed all study procedures. Volunteers were recruited from those who completed a comparative pharmacology study of the behavioral and cognitive effects of three oral doses of psilocybin (10, 20, and 30 mg/70 kg) and one oral dose of dextromethorphan (400 mg/70 kg) under blinded conditions (Barrett et al., 2018; Carbonaro et al., 2018). At least one month elapsed between final psychoactive drug administration in the parent study and the beginning of the current study, and thus there was no risk of carry-over of drug effects from the parent study to the current study, as the half-lives of psilocybin and dextromethorphan are roughly 2–4 h. All volunteers underwent medical and psychiatric screening, including physical examination, electrocardiogram, routine medical blood tests, and the Structured Clinical Interview for Diagnosis (SCID-IV) (First et al., 1997) before the current study. Individuals were excluded from participation if they had a history of substance dependence according to DSM-IV-TR criteria (excluding nicotine and caffeine), were pregnant or nursing, had a current significant medical condition, had a current DSM-IV Axis-I disorder, had a personal or immediate family history of schizophrenia, bipolar affective disorder, delusional disorder, paranoid disorder, or schizoaffective disorder, were taking any centrally-acting serotonergic drug, or antidepressant or antipsychotic medication, had not well-tolerated psilocybin administration in the parent trial (Carbonaro et al., 2018), met exclusionary criteria for MRI or PET (including implanted medical devices that are contraindicated for MRI, claustrophobia incompatible with MRI or PET procedures, and current-year radiation exposure that, coupled with study procedures, would exceed safe limits), were pregnant, nursing, or were positive for drugs of abuse (including alcohol). Written informed consent was obtained according to procedures established by the Institutional Review Board and the Radioactive Drug Research Committee (RDRC) of the Johns Hopkins University School of Medicine.

### Procedures

Prior to the current study, and consistent with guidelines for the safe administration of hallucinogen drugs in research (Johnson et al., 2008), participants had established good rapport with two study team members who served as monitors for all five experimental drug administration sessions in the parent study (Carbonaro et al., 2018). During the initial consent process for the current study, volunteers were informed that they had received a 10 mg/70 kg dose of psilocybin in the parent study, and they would receive that dose of psilocybin again in the current study. Volunteers also reviewed the subjective reports that they had provided of their experience with the 10 mg/70 kg dose of psilocybin in the parent study. This was done to orient the volunteer to the strength and character of drug effect that they might reasonably expect to experience in the current study.

Volunteers completed an MRI scan to obtain a structural brain image for use as a screening tool to identify and exclude those with significant structural abnormalities, and to aid in PET image registration. Participants also completed resting-state functional MRI that was used for group-specific brain parcellation. A baseline (pre-treatment) PET scan with [^11^C] MDL 100,907 was completed at least two days before the blocking scan (a PET scan after pretreatment with psilocybin). At least one of the participant's monitors from the parent study was present during all procedures in the current study. Participants were instructed to consume a low-fat breakfast and their usual amount of caffeine before arriving at the laboratory on the morning of the psilocybin administration, and they were told to refrain from using any drugs while enrolled in the study. Before the baseline and blocking PET scans, all participants underwent a brief physical examination, and urine was tested for a panel of commonly abused drugs and for pregnancy in female participants. Negative results were required to proceed. During PET scan, participants were instructed to turn their attention inward while listening to a standard playlist of music that has been provided in previous studies (Griffiths et al., 2006, 2011, 2016, 2018; Johnson et al., 2014; Carbonaro et al., 2018; Barrett et al., 2020; Davis et al., 2021). During PET scans, participants were in continuous contact with study staff, including a monitor from their experimental sessions in the parent study. Participants were administered a 10 mg/70 kg body weight oral dose of psilocybin 80 min before radiotracer injection for the blocking scan, in order to assess psilocybin binding proximate in time to the estimated C_max_ (Passie et al., 2002; Brown et al., 2017).

### MRI Acquisition and Preprocessing

A high-resolution structural image (MPRAGE; 1 mm slice thickness) and resting-state blood-oxygenation level-dependent data (180 volumes of two-dimensional echo-planar imaging sequences, or EPIs; 3 x 3 mm in-plane resolution, 3 mm slice thickness, 1 mm gap, 30 slices, TR = 2.46 s, total scan time = 7 min 22 s) were collected for each participant using a 3T Siemens Skyra whole-body scanner with a 32-channel head coil at the Center for Translational Molecular Imaging of the Johns Hopkins University School of Medicine. The MPRAGE was segmented into gray matter, white matter, and cerebrospinal fluid (CSF) masks, and then normalized to the MNI152 template through indirect normalization, which implements non-linear registration through registration of gray-matter masks to a tissue probability map for the MNI152 template (Ashburner and Friston, 2005). Resting state data were preprocessed with slice timing correction, motion correction, co-registration to the MPRAGE image, MPRAGE normalization to the MNI template using the normalized mutual information algorithm (Ashburner and Friston, 2005), propagation of MPRAGE normalization parameters to co-registered resting-state images, smoothing with a 6 mm full-width at half maximum (FWHM) kernel, motion scrubbing (Power et al., 2012), linear detrending, regression of nuisance variables including motion (6 motion parameters) as well as the first five principle components of average signal from ventricles and white matter (Behzadi et al., 2007), and band-pass filtering between 0.008 and 0.09 Hz. Preprocessing was conducted using Statistical Parametric Mapping version 12 (SPM12; http://www.fil.ion.ucl.ac.uk/spm), custom scripts in MATLAB (The MathWorks Inc.), and publicly available scripts from the Cognitive Affective Neuroscience Laboratory at the University of Colorado, Boulder (http://github.com/canlab).

Preprocessed resting-state data were clustered into 200 parcels, or regions of interest (ROIs), using spectral clustering of the average (Fisher-transformed, averaged across-subjects) between-voxel gray matter Pearson correlation matrix (Craddock et al., 2012). These ROIs were then used for PET ROI analysis.

### PET Acquisition, Preprocessing, and Quantification

PET scans were acquired in the Russell H. Morgan Department of Radiology, Johns Hopkins Hospital, using a GE Advance PET scanner. A thermoplastic mask was modeled to each participant's face and used to reduce head motion during the PET study. The radiotracer [^11^C]MDL 100,907 was synthesized using C-11 methylation (Wong et al., 2008) and was used to measure availability of the 5-HT_2A_ receptor. [^11^C]MDL 100,907 dynamic scanning began immediately following a 20.46 mCi ± 0.55 mCi radiotracer injection (specific activity at the baseline scan: 10,432 mCi/μmole ± 3,109, specific activity at the blocking scan: 10,358 mCi/μmole ± 3,224) and lasted for 90 min, and scans were obtained using a 30-frame acquisition protocol (framing sequences: 4 x 15, 4 x 30, 3 x 60, 2 x 120, 5 x 240, and 12 x 300 s). All PET images (image size 128 ×128, pixel size 2 ×2 mm, slice thickness 4.25, 4.5 mm FWHM at the center of the field of view) were reconstructed using filtered back projection with decay and attenuation correction (Zhou et al., 2010). The summed 90-min dynamic PET images were used for PET-to-PET registration and MRI-to-PET co-registration using SPM and MATLAB. The MRI images and dynamic PET scans following treatment were registered to the baseline PET scans for each subject (Martin-Facklam et al., 2013). Co-registered images were normalized to the MNI template through the MRI image using the normalized mutual information algorithm (Ashburner and Friston, 2005).

The ROI time activity curves (TACs) were calculated by applying the ROIs generated from resting-state data to dynamic PET images. The binding potential (BP_ND_) (Koeppe et al., 1991; Innis et al., 2007), an index of tracer-specific binding to receptors, was estimated by simultaneously fitting a simplified reference tissue model (SRTM of 3 parameters R_1_, k_2REF_, BP_ND_) to all ROI TACs with k_2REF_ coupling (Zhou et al., 2007). Cerebellum was used as reference tissue input, as the concentration of 5-HT_2A_ receptors in this region is negligible, and this region has been validated as a reference region for [^11^C] MDL 100,907 (Hall et al., 2000; Talbot et al., 2012). The ROI percent occupancy (Martin-Facklam et al., 2013) was calculated as:


$$\begin{array}{l} Occ\left(\%\right)=\;100*\frac{BP_{ND}\left(baseline\right)-\;BP_{ND}\left(blocking\right)}{BP_{ND}\left(baseline\right)} \end{array}$$

The ROIs exhibiting low binding potential (< 0.2), low distribution volume ratio (< 1.2), or impossible occupancy (< 0% or > 100%) were discarded (including striatum and basal ganglia) and the SRTM model was re-fit with the remaining ROIs. 137 ROIs (listed in Table 1) in total were retained for analysis. BP at baseline and occupancy by psilocybin were then calculated for each retained ROI.

**Table 1:** Baseline binding potential and occupancy by psilocybin of 5-HT_2A_ receptors in each region of interest for each subject.

	**5-HT_2A_** **Receptor Binding Potential**					**5-HT_2A_** **Receptor Occupancy (%)**				
	**(BP_ND_) at Baseline**					**by Psilocybin**				
**ROIs**	**S1**	**S2**	**S3**	**S4**	**Mean**	**S1**	**S2**	**S3**	**S4**	**Mean**
l angular gyrus	0.79	0.84	3.42	3.94	2.25	85.34	74.57	90.51	48.46	74.72
l intraparietal sulcus	0.84	1.05	2.56	2.50	1.74	77.22	63.94	82.71	59.99	70.97
r angular gyrus	0.82	1.32	2.56	2.97	1.92	61.88	65.53	83.98	70.52	70.47
superior parietal gyrus	0.82	0.66	1.79	2.34	1.40	72.57	49.38	81.95	70.14	68.51
l precentral gyrus	0.77	0.59	2.47	2.17	1.50	80.61	61.62	79.99	51.34	68.39
r intraparietal sulcus	0.74	1.30	2.19	2.23	1.61	72.12	59.76	70.93	60.90	65.93
sgACC	1.00	1.21	2.25	2.50	1.74	73.25	62.10	77.02	40.10	63.12
r postcentral gyrus	0.90	0.94	1.36	1.47	1.17	71.15	39.53	67.22	65.07	60.74
l posterior SFG	1.06	0.94	2.01	3.36	1.84	76.95	43.10	75.84	46.16	60.51
l postcentral gyrus	0.81	1.12	2.02	1.92	1.47	61.20	56.36	69.11	53.33	60.00
r dorsal lateral parietal	0.76	1.20	1.80	2.53	1.57	72.29	49.88	61.40	52.95	59.13
r precentral gyrus	0.79	0.98	1.44	1.33	1.14	66.85	43.46	68.03	57.88	59.05
l sensorimotor	1.09	1.14	1.67	1.75	1.41	64.19	44.41	71.94	46.26	56.70
l posterior MFG	0.85	0.81	2.46	1.73	1.46	65.23	56.51	63.32	37.97	55.76
l lateral sensorimotor	0.79	0.90	1.62	1.19	1.12	64.78	41.03	58.14	54.75	54.68
r premotor	1.02	1.18	1.75	1.56	1.38	55.86	40.59	65.86	54.01	54.08
l postcentral gyrus	0.97	1.24	1.82	1.62	1.41	57.08	43.24	60.68	41.41	50.60
l dorsal mid-insula	1.11	1.46	2.03	1.91	1.63	52.70	43.11	53.68	50.79	50.07
cuneus/calcarine sulcus	1.19	1.30	1.66	2.05	1.55	55.76	37.26	51.64	52.91	49.39
r posterior SFG	1.00	1.65	1.45	2.43	1.63	70.32	24.59	61.33	41.15	49.35
l premotor	1.11	1.37	1.58	2.16	1.55	63.16	30.94	62.22	40.89	49.30
l ventral sensorimotor	0.69	0.94	1.62	1.21	1.12	55.59	46.78	63.43	28.28	48.52
r posterior IFG	0.81	1.41	1.15	1.36	1.18	50.59	26.28	50.40	63.35	47.66
l SMA	1.13	1.71	1.59	2.10	1.63	57.30	29.25	57.60	45.84	47.50
r dorsal mid-insula	1.19	2.01	2.08	1.95	1.81	52.19	37.25	55.55	41.92	46.73
l hippocampus	0.52	0.81	1.00	0.64	0.74	49.43	22.87	63.69	50.75	46.68
r STG (BA41)	1.06	1.72	1.69	1.73	1.55	60.54	31.56	52.85	41.32	46.57
medial sensorimotor	0.97	1.30	1.78	1.20	1.31	48.95	37.45	51.96	47.12	46.37
r posterior SFS	1.09	1.91	1.60	1.77	1.59	49.14	29.26	49.97	53.75	45.53
l middle occipital gyrus	1.04	1.44	1.73	1.64	1.46	51.63	32.43	52.81	44.79	45.42
dorsal motor	1.08	1.22	1.72	1.20	1.31	51.42	33.74	49.40	45.52	45.02
r sensorimotor	0.84	1.89	1.53	1.33	1.40	29.64	28.55	60.38	59.46	44.51
l STG (BA41)	1.32	2.17	2.10	2.07	1.91	42.63	32.75	55.66	45.77	44.20
l medial temporal pole	0.81	1.39	1.06	1.55	1.20	48.08	32.74	58.90	36.31	44.01
l IFS	0.89	1.43	1.75	1.50	1.39	53.00	36.23	46.26	39.79	43.82
l posterior IFG	0.58	0.98	1.24	1.14	0.98	47.41	37.73	54.17	35.52	43.71
l postcentral gyrus	0.61	0.51	0.96	0.66	0.69	22.86	22.87	54.59	73.43	43.44
r postcentral gyrus	1.08	1.52	1.24	1.36	1.30	50.35	20.48	49.61	53.29	43.43
r posterior MFG	1.17	2.03	1.74	1.92	1.71	48.24	26.92	44.14	53.18	43.12
r dorsal posterior insula	1.28	1.96	1.93	1.79	1.74	50.60	25.97	55.98	39.22	42.94
l dorsal posterior insula	1.28	2.11	1.90	1.66	1.74	45.72	28.79	46.20	50.79	42.88
l posterior STS	0.84	1.62	1.56	1.60	1.40	45.41	33.85	46.56	44.83	42.66
l SFG	1.08	1.34	1.76	1.78	1.49	49.29	33.09	49.02	36.18	41.90
l dorsal precuneus	0.90	1.41	1.64	1.40	1.34	42.49	31.21	43.68	49.66	41.76
r ventral anterior insula	0.57	1.05	0.93	0.82	0.84	50.26	28.07	40.45	47.81	41.65
r middle occipital gyrus	1.01	1.53	1.33	1.26	1.28	41.81	24.06	48.63	51.16	41.42
l posterior MTG	1.05	1.67	1.76	1.67	1.54	44.10	35.34	48.35	37.72	41.38
l occipital pole	0.48	0.75	0.46	1.03	0.68	30.45	36.35	42.00	56.02	41.21
l posterior MFG	0.94	1.74	1.93	1.63	1.56	46.15	39.92	40.82	37.36	41.06
l SMG	0.91	1.65	1.98	1.67	1.55	42.20	35.75	46.22	39.90	41.02
l postcentral gyrus	1.03	1.85	1.80	1.57	1.56	44.19	32.14	43.56	43.32	40.80
l cuneus	1.30	1.81	2.27	1.83	1.80	53.00	27.84	41.18	40.75	40.69
r mid post OFC	0.82	1.45	1.21	1.31	1.19	57.95	24.15	43.08	36.66	40.46
l anterior SFG	1.07	1.78	1.76	1.75	1.59	42.83	30.44	48.89	38.36	40.13
l IFG (pars triangularis)	1.03	1.65	1.85	1.64	1.54	41.93	40.56	42.77	35.24	40.13
l mid post OFC	0.74	1.06	1.04	1.22	1.01	58.98	15.92	55.56	29.99	40.11
r SFG	1.09	1.63	1.78	1.81	1.58	44.80	27.21	43.98	43.32	39.83
r middle STG	0.90	1.68	1.28	1.38	1.31	46.39	11.12	43.89	57.30	39.67
r medial temporal pole	0.87	1.63	1.19	1.51	1.30	49.79	15.09	53.03	39.93	39.46
r occipital pole	0.75	1.15	0.78	1.42	1.03	42.16	24.66	39.38	50.84	39.26
r dorsal precuneus	0.90	1.43	1.63	1.25	1.30	42.12	27.54	43.51	43.45	39.15
l inferior occipital gyrus	0.86	1.30	0.90	1.41	1.12	44.99	16.32	49.73	44.98	39.00
r IFS	1.11	1.73	1.75	1.67	1.56	40.03	28.28	40.29	46.90	38.88
l MFG	1.24	1.68	1.98	1.80	1.68	45.60	35.54	43.36	30.92	38.86
l medial orbital gyrus	1.33	2.28	1.56	1.94	1.78	41.16	21.10	49.44	43.06	38.69
r angular gyrus	0.91	1.93	1.59	1.44	1.46	35.70	26.67	42.75	48.00	38.28
l angular gyrus	1.11	1.88	2.13	1.69	1.70	42.00	35.93	40.16	33.89	38.00
r STG	0.83	1.84	1.35	1.25	1.32	39.08	17.24	40.39	53.28	37.50
r MFG	1.27	2.09	1.71	1.94	1.75	46.93	15.31	44.28	42.90	37.36
r SMG	0.97	1.75	1.52	1.16	1.35	20.65	24.90	43.61	60.18	37.34
l STS	1.16	1.96	1.64	1.61	1.59	34.31	27.20	49.86	36.99	37.09
r posterior MTG	0.86	1.67	1.10	1.46	1.27	33.64	16.95	41.74	55.42	36.94
r medial orbital gyrus	1.26	1.97	1.49	2.11	1.71	53.94	11.86	41.92	39.70	36.86
r posterior MFG	1.16	2.24	1.66	1.65	1.68	45.75	23.72	29.57	48.26	36.82
l SFS	1.44	1.86	1.63	1.84	1.69	38.61	21.03	51.05	36.49	36.79
r IFG (pars triangularis)	1.23	2.10	1.78	1.87	1.74	40.54	16.01	43.73	46.25	36.63
medial OFC	1.36	2.37	1.82	2.00	1.89	42.60	23.91	39.82	39.34	36.42
r hippocampus	0.37	0.76	0.67	0.57	0.59	49.72	16.52	41.18	37.67	36.27
l dorsal anterior insula	1.27	2.04	1.80	1.77	1.72	44.68	23.90	37.02	39.38	36.25
r dorsal anterior insula	1.52	2.30	1.70	1.94	1.86	46.33	20.27	40.61	36.51	35.93
r perirhinal	0.80	1.58	1.09	1.28	1.19	50.16	7.75	47.85	37.72	35.87
r inferior lingual gyrus	0.87	1.36	1.24	1.19	1.16	45.62	18.77	31.95	45.84	35.54
posterior cuneus	1.61	2.00	2.30	2.31	2.05	38.28	25.41	34.47	43.51	35.42
dorsal medial precuneus	1.23	2.09	1.85	1.69	1.72	39.22	25.20	33.00	44.12	35.39
r frontal polar gyrus	1.15	2.10	1.74	1.72	1.68	36.56	26.73	38.34	39.90	35.38
l parieto-occipital fissure	1.38	1.93	1.73	1.69	1.68	37.33	24.06	36.91	43.22	35.38
r STS	1.24	2.22	1.59	1.85	1.73	40.60	9.59	44.07	46.33	35.15
l middle ITS	1.19	2.09	1.75	2.01	1.76	41.06	23.91	40.33	34.00	34.82
l IFG	1.15	1.74	1.59	1.62	1.53	41.50	17.85	43.11	36.33	34.70
l anterior MFG	1.33	2.17	1.75	1.82	1.77	39.73	23.34	39.37	36.09	34.63
preSMA	1.16	1.91	1.84	1.63	1.63	40.96	20.73	38.10	38.22	34.50
r pgACC	1.22	2.12	1.78	1.71	1.71	41.75	18.81	34.87	41.84	34.32
l frontal polar gyrus	1.11	2.27	1.65	1.58	1.65	36.14	22.62	35.49	42.12	34.09
r anterior SFG	1.03	1.82	1.64	1.64	1.53	42.38	18.02	35.16	40.01	33.89
l posterior ITS	1.14	1.98	1.66	1.90	1.67	27.39	25.78	42.15	38.35	33.42
l inferior lingual gyrus	0.98	1.35	1.22	1.24	1.20	37.91	15.82	42.82	36.95	33.37
r SFS	1.16	2.13	1.42	1.84	1.64	44.59	11.13	33.32	43.93	33.24
r posterior STS	1.34	2.55	1.87	1.88	1.91	36.91	14.85	33.69	46.80	33.06
r cuneus	1.34	1.98	2.24	1.70	1.82	28.54	24.93	35.76	42.70	32.98
l anterior STS	1.08	2.18	1.54	1.45	1.56	36.74	17.93	38.00	38.51	32.79
r anterior MFG	1.23	2.39	1.66	1.78	1.77	36.04	21.84	28.49	44.74	32.78
l anterior orbital gyrus	1.28	1.83	1.24	1.80	1.54	43.83	0.00	47.54	39.22	32.65
r parieto-occipital fissure	1.36	1.72	1.61	1.65	1.58	34.76	15.89	34.21	44.72	32.39
SMA	1.19	2.24	1.70	1.74	1.72	39.12	16.50	32.09	40.62	32.08
l pgACC	1.22	2.13	1.92	1.83	1.78	35.99	25.26	35.12	31.22	31.90
r middle ITS	1.06	2.20	1.27	1.85	1.59	40.58	3.91	35.02	46.15	31.42
r posterior lingual gyrus	0.80	1.20	0.93	1.21	1.03	38.07	15.68	35.77	36.00	31.38
l anterior STG	0.81	1.79	0.83	1.01	1.11	21.55	13.75	43.04	46.99	31.33
l anterior temporal pole	0.71	1.55	1.04	1.04	1.08	32.17	5.49	45.35	41.58	31.15
r IFG (pars opercularis)	0.86	1.75	0.93	1.14	1.17	31.04	0.00	30.48	62.07	30.90
r posterior orbital gyrus	1.26	1.75	1.13	1.85	1.50	47.14	0.00	41.65	34.10	30.72
r temporal pole	0.96	1.57	0.88	1.75	1.29	45.07	2.84	43.72	29.66	30.32
l ITG	0.50	1.13	0.78	1.19	0.90	40.31	17.56	32.53	29.82	30.06
r ventral mid-insula	0.96	1.77	1.20	1.24	1.29	36.39	6.55	34.22	42.84	30.00
r anterior STS	0.95	1.88	1.32	1.37	1.38	38.23	10.33	30.20	41.04	29.95
PCC	1.40	2.36	1.73	1.79	1.82	36.08	10.39	33.52	37.84	29.46
r inferior occipital gyrus	0.81	0.98	0.62	0.91	0.83	16.31	0.00	41.66	59.85	29.45
r posterior ITG	0.97	1.55	0.95	1.23	1.17	29.61	1.34	32.98	50.23	28.54
l anterior ITG	1.18	2.05	1.24	1.87	1.58	35.48	3.02	43.01	32.11	28.41
l posterior ITG	0.76	1.59	1.03	1.27	1.17	32.44	10.01	35.09	35.40	28.23
l inferior occipital gyrus	0.92	1.31	0.96	1.30	1.12	31.30	6.62	34.57	39.96	28.11
l posterior lingual gyrus	1.16	1.76	1.02	1.51	1.36	28.28	12.20	31.62	40.30	28.10
r fusiform gyrus	0.90	1.11	1.14	1.27	1.11	29.89	14.12	39.69	28.19	27.97
midcingulate	1.10	2.06	1.24	1.46	1.46	42.84	0.00	28.78	40.26	27.97
r posterior ITS	0.93	1.87	0.96	1.41	1.29	25.91	0.00	35.61	49.71	27.80
r anterior ITG	1.07	2.28	1.00	1.60	1.49	38.85	0.00	32.59	39.42	27.72
r lateral orbital gyrus	1.08	2.43	1.32	1.57	1.60	34.50	0.00	31.98	42.53	27.25
dACC	1.11	2.27	1.43	1.24	1.51	29.70	9.89	30.56	38.16	27.08
medial precuneus	1.22	1.95	1.38	1.47	1.51	33.65	9.62	23.60	35.01	25.47
l lateral orbital gyrus	1.22	2.46	1.57	1.68	1.73	26.26	0.64	30.46	43.25	25.15
l IFG (pars opercularis)	0.97	1.69	1.21	1.27	1.28	30.16	7.71	28.96	33.27	25.02
l fusiform gyrus	0.54	0.65	0.53	0.62	0.58	35.33	10.02	30.58	23.00	24.73
posterior dACC	1.13	2.18	1.23	1.12	1.41	23.55	0.00	33.26	40.20	24.25
r anterior fusiform	0.54	0.70	0.61	0.71	0.64	25.83	3.59	32.59	34.62	24.16
l IFG pars orbitalis	1.06	1.98	1.19	1.59	1.46	30.43	0.00	26.10	32.20	22.19
l putamen	0.06	0.27	0.26	−0.04	0.14	7.70	3.87	43.55	28.29	20.85
r IFG (pars orbitalis)	0.92	2.12	0.69	1.19	1.23	13.42	0.00	14.45	51.48	19.84

*Data are sorted in descending order by mean occupancy by psilocybin. S1-S4: subject 1 through subject 4; S1 and S4 were male, and S2 and S3 were female. L, left; r, right; SFG, superior frontal gyrus; SFS, superior frontal sulcus; MFG, middle frontal gyrus; IFS, inferior frontal sulcus; IFG, inferior frontal gyrus; OFC, orbitofrontal cortex; SMA, supplementary motor area; STG, superior temporal gyrus; STS, superior temporal sulcus; MTG, middle temporal gyrus; ITS, inferior temporal sulcus; ITG, inferior temporal gyrus; SMG, supramarginal gyrus; ACC, anterior cingulate cortex; PCC, posterior cingulate cortex*.

*Mean across subjects for 5-HT_2A_ receptor availability and occupancy by psilocybin for each region of interest are shaded to aid in readability*.

## Results

**Figure 1:**
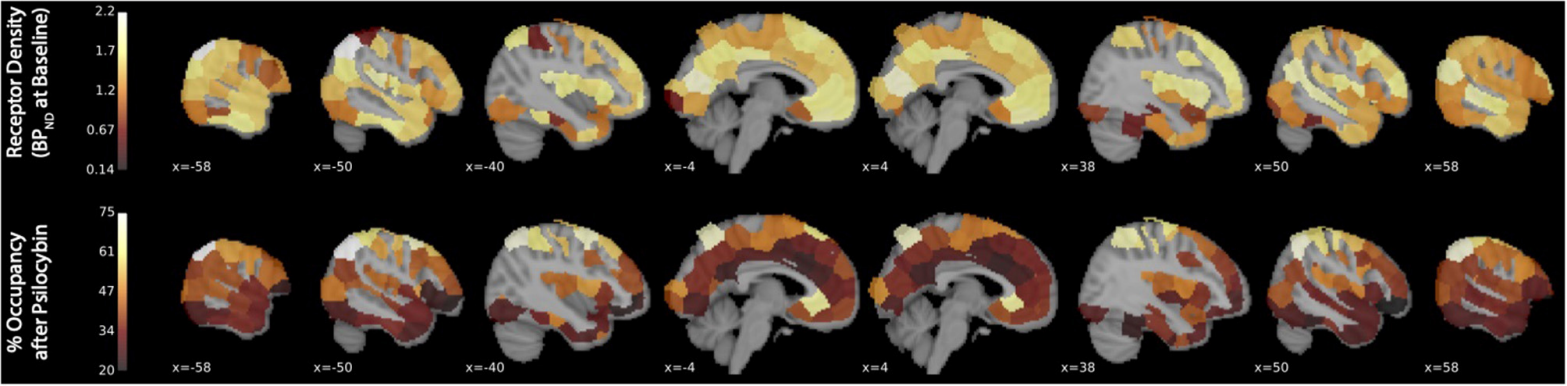
5-HT_2A_ receptor binding potential (BP_ND_) at baseline and 5-HT_2A_ receptor occupancy by psilocybin. The x-coordinate of each sagittal brain slice in Montreal Neurological Institute coordinates is presented in the lower-left-hand corner of each panel. The color bar identifying the range of plotted values is presented on the left-hand side of each plot. BP_ND_ is presented in normalized values relative to the reference region (cerebellum). Occupancy by psilocybin is presented in percentages of total possible occupancy.

**Figure 2:**
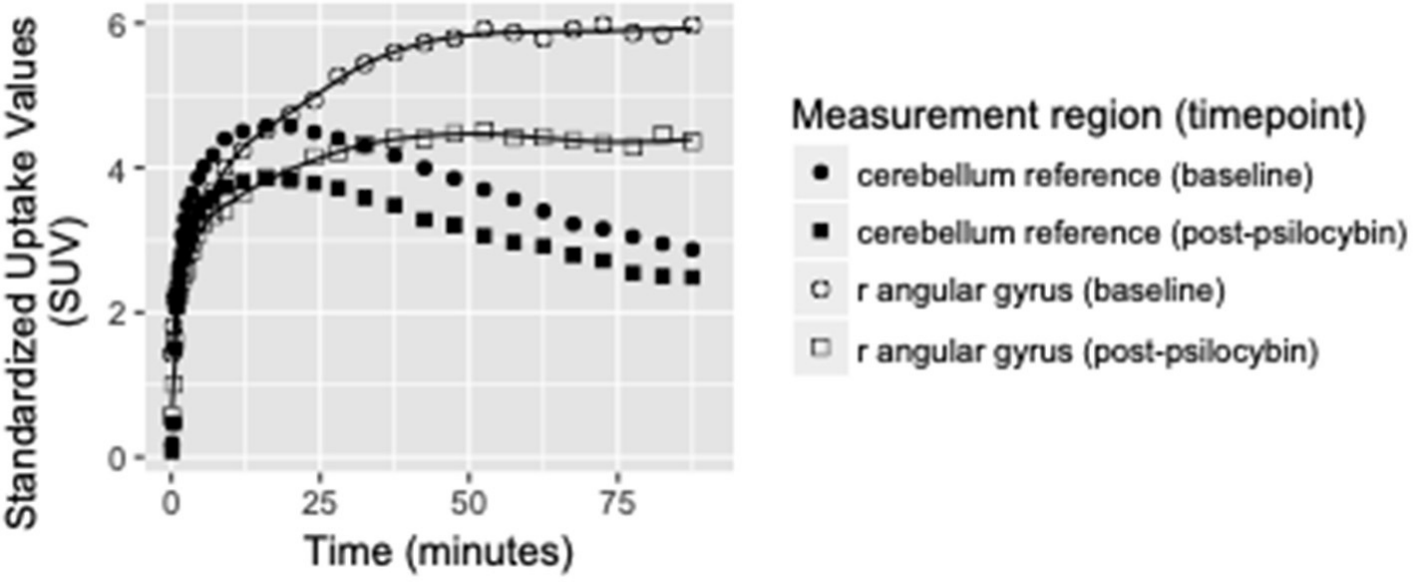
Time activity curves at baseline and post-psilocybin time points for the right angular gyrus and the cerebellum reference region in a representative volunteer (S4, a 28-year-old male). Differences between baseline and post-psilocybin standardized uptake values demonstrate substantial blocking of [^11^C]MDL 100,907 by psilocybin in the angular gyrus, but not in the reference region (as expected). Time activity curves for the right angular gyrus are representative of time activity curves for others regions with highest occupancy by psilocybin (including left angular gyrus, bilateral intraparietal sulcus, and subgenual anterior cingulate cortex).

Binding potential (BP_ND_) of [^11^C]MDL 100,907 at baseline (reflecting available 5-HT_2A_ receptor binding sites) and 5-HT_2A_ receptor occupancy by psilocybin for each individual is presented in Table 1. Average baseline 5-HT_2A_ receptor binding potential (BP_ND_) was 1.44 (± 0.33 SD), and average 5-HT_2A_ receptor occupancy across all ROIs was 39.5% (± 10.9% SD). Group-averaged 5-HT_2A_ BP_ND_ at baseline and 5-HT_2A_ receptor occupancy by psilocybin are represented in Figure 1. Brain regions demonstrating greatest 5-HT_2A_ receptor occupancy by psilocybin (between 63.12 and 74.72% occupancy) included bilateral angular gyrus, bilateral intraparietal sulcus, precentral gyrus, and subgenual anterior cingulate cortex. Time activity curves for the right angular gyrus and the reference region (cerebellum) in a representative volunteer are presented in Figure 2.

## Discussion

The 5-HT_2A_ receptor is implicated in a number of psychiatric disorders, most notably mood disorders and psychosis (Sibille et al., 1997; Marek et al., 2003; Frokjaer et al., 2008). Recent brain imaging studies have shown 5-HT_2A_ receptor occupancy in humans by psilocybin (Madsen et al., 2019; Stenbæk et al., 2021). Studies have also shown psilocybin, as well as other classic hallucinogens, to alter activity and connectivity in primary sensory regions (Kaelen et al., 2016; Roseman et al., 2016; Barrett et al., 2017; Mason et al., 2020; Preller et al., 2020) and regions of the default mode network (Carhart-Harris et al., 2012, 2014, 2016b; Mason et al., 2020). These are brain regions that both densely express 5-HT_2A_ receptors (Gründer et al., 1997; Hall et al., 2000; Kakiuchi et al., 2000) and are implicated in the pathophysiology of mood disorders (Greicius, 2008) and addiction (Sutherland et al., 2012). Further studies have suggested that alteration of brain function in these regions may be a mechanism of therapeutic effects of psychedelics (Carhart-Harris et al., 2017; Sampedro et al., 2017; Doss et al., 2021).

The current report provides additional evidence for substantial psilocybin occupancy of 5-HT_2A_ receptors throughout the cortex in humans, using a different 5-HT_2A_ ligand than had previously been used (Madsen et al., 2019; Stenbæk et al., 2021). All 137 ROIs that were retained for analysis are reported, since average occupancy in each of these regions ranged from 19.84 to 74.72%, and the magnitude of occupancy well-exceed the test-retest variability (7-11%) of [^11^C] MDL 100,907 (Talbot et al., 2012). Three of the regions with greatest occupancy by psilocybin are within the default mode network (sgACC and bilateral angular gyrus), but individual occupancy levels varied widely between individuals in these ROIs (e.g., between 48.5 and 90.5% in the L angular gyrus; Table 1).

A clear limitation of this pilot study is the small sample size. Alternate explanations for individual variability in subjective effects of psilocybin may be genetic polymorphisms related to 5-HT_2A_ receptor expression and function, and/or differences in the bioavailability of psilocybin and its active metabolite psilocin, however we did not test for these factors in this limited sample. Future studies, including controlled measurement of plasma levels of psilocin, will determine whether some or all of these factors are involved.

The current report demonstrates widespread and substantial occupancy of cortical 5-HT_2A_ receptors by psilocybin. Future investigations of the relationship between 5-HT_2A_ receptor occupancy and both subjective effects of psilocybin and a wider array of other brain-derived measures (e.g., functional connectivity, task-based neural responses, pharmacokinetic and pharmacodynamic measures) may yield important insights into the mechanisms underlying individual differences in both the acute response to psychedelics and enduring therapeutic responses to psychedelics in patient populations.

## Data Availability Statement

The datasets presented in this article are not readily available because data availability is limited to qualified researchers pending institutional agreements. Requests to access the datasets should be directed to FB, fbarrett@jhmi.edu.

## Ethics Statement

The studies involving human participants were reviewed and approved by Johns Hopkins Medicine Institutional Review Board. The patients/participants provided their written informed consent to participate in this study.

## Author Contributions

FB and GS conceived of the study. FB, TC, JR, GS, DW, and RG contributed to the study design. FB, TC, and JR carried out the research. RG supervised experimental drug administration procedures. FB and YZ designed and carried out the analysis. FB wrote the manuscript, and all authors edited and provided feedback on the manuscript.

## Funding

This publication was made possible by the Johns Hopkins Institute for Clinical and Translational Research (ICTR) which is funded in part by Grant Number UL1 TR 001079 from the National Center for Advancing Translational Sciences (NCATS) a component of the National Institutes of Health (NIH), and NIH Roadmap for Medical Research. Its contents are solely the responsibility of the authors and do not necessarily represent the official view of the Johns Hopkins ICTR, NCATS, or NIH. This study was also supported in part by grants from the National Institutes of Health: R01DA03889, T32DA07209, R03DA042336, 1R01DA042094, R01AA023483, R01MH107197, R01MH10719705, and R01DA042094. TC is now an employee of the U.S. Drug Enforcement Agency (DEA); however, the views presented in this article do not necessarily reflect those of the DEA and no official support or endorsement of this article by the DEA is intended or should be inferred.

## Conflict of Interest

RG is on the Board of Directors of the Heffter Research Institute. YZ is employed by the company United Imaging Intelligence. The remaining authors declare that the research was conducted in the absence of any commercial or financial relationships that could be construed as a potential conflict of interest.

## Publisher's Note

All claims expressed in this article are solely those of the authors and do not necessarily represent those of their affiliated organizations, or those of the publisher, the editors and the reviewers. Any product that may be evaluated in this article, or claim that may be made by its manufacturer, is not guaranteed or endorsed by the publisher.
